# Supplementary material for: Using machine learning for detection of Parkinson’s disease and mild cognitive impairment
Source: PLoS One. 2025 Nov 19;20(11):e0335541. doi: 10.1371/journal.pone.0335541 (PMC12629485; doi:10.1371/journal.pone.0335541)
Supplement: S1a Table — Abbreviations: PD; Parkinson’s Disease, HC; Healthy controls, NC; Normal Cognition; MCI; Mild Cognitive Impairment; DaT; DaT-SPECT; SBR; Striatal Binding Ratio; α-syn; Alpha-synuclein; Aβ42; Beta-amyloid-42; t-tau; total-tau; p-tau; phosphorylated-tau-181; NfL; neurofilament light; Conc.; Concentration; pg; picogram; ml; millilitre, mo; month. All variables are reported with Mean (Standard Deviation). Sample sizes are reported in Table 2. (PDF) [file pone.0335541.s001.pdf]

S1a Table: Biomarker Analysis

Measurement	Biomarker	PD	HC	*p*-value	t	Degrees of Freedom
DaT Baseline SBR	Left Caudate	1.87 (0.57)	2.96 (0.59)	**< .001**	**14.705**	**223.55**
	Right Caudate	1.87 (0.60)	2.92 (0.58)	**< .001**	**13.885**	**216.22**
	Left Putamen	0.77 (0.34)	2.12 (0.54)	**< .001**	**24.47**	**249.26**
	Right Putamen	0.80 (0.32)	2.12 (0.54)	**< .001**	**24.379**	**246.18**
	Left Anterior Putamen	1.23 (0.43)	2.58 (0.56)	**< .001**	**21.629**	**246.67**
	Right Anterior Putamen	1.27 (0.42)	2.59 (0.58)	**< .001**	**21.161**	**250.11**
CSF Baseline Conc. (pg/ml)	α-syn	1544.73 (707.91)	1718.58 (782.78)	0.070	1.822	230.23
	Aβ42	805.43 (326.78)	929.21 (363.09)	**0.006**	**2.761**	**225.62**
	t-tau	176.33 (63.33)	192.51 (80.54)	0.077	1.775	244.66
	p-tau	15.71 (5.88)	17.55 (8.72)	0.054	1.936	234.99
	NfL	117.68 (69.84)	100.37 (55.79)	0.100	-1.659	117.86
CSF Secondary Conc. (pg/ml)	α-syn	1517.14 (639.98)	1791.28 (768.96)	**0.003**	**3.053**	**238.49**
	Aβ42	752.79 (352.89)	917.32 (341.38)	**< .001**	**3.609**	**209.06**
	t-tau	186.62 (78.68)	202.82 (91.65)	0.136	1.496	237.9
	p-tau	16.35 (6.87)	18.24 (9.21)	0.073	1.803	233.03
	NfL	161.98 (144.81)	108.70 (55.00)	**0.006**	**-2.823**	**77.148**
CSF Rate of Change (pg/(ml*mo))	α-syn	-0.37 (15.57)	2.86 (22.26)	0.179	1.347	246
	Aβ42	1.10 (8.11)	0.13 (9.71)	0.402	-0.839	232.18
	t-tau	0.20 (1.25)	0.18 (1.46)	0.909	-0.115	237.94
	p-tau	0.01 (0.09)	0.01 (0.13)	0.948	-0.066	232.8
	NfL	1.28 (2.92)	0.19 (1.39)	0.007	-2.790	84.567

Abbreviations: PD; Parkinson's Disease, HC; Healthy controls, NC; Normal Cognition; MCI; Mild Cognitive Impairment; DaT; DaT-SPECT; SBR; Striatal Binding Ratio; α-syn; Alpha-synuclein; Aβ42; Beta-amyloid-42; t-tau; total-tau; p-tau; phosphorylated-tau-181; NfL; neurofilament light; Conc.; Concentration; pg; picogram; ml; millilitre, mo; month.

All variables are reported with Mean (Standard Deviation). Sample sizes are reported in Table 2.
