# Supplementary material for: Using machine learning for detection of Parkinson’s disease and mild cognitive impairment
Source: PLoS One. 2025 Nov 19;20(11):e0335541. doi: 10.1371/journal.pone.0335541 (PMC12629485; doi:10.1371/journal.pone.0335541)
Supplement: S1b Table — Abbreviations: PD – Parkinson’s Disease, HC – Healthy controls, NC – Normal Cognition; MCI – Mild Cognitive Impairment; DaT – DaT-SPECT; SBR – Striatal Binding Ratio; α-syn – Alpha-synuclein; Aβ42 – Beta-amyloid-42; t-tau – total-tau; p-tau – phosphorylated-tau-181; NfL – neurofilament light; Conc. - Concentration; pg – picogram; ml – millilitre; mo – month. All variables are reported with Mean (Standard Deviation). * Sample sizes are reported in Table 2. (PDF) [file pone.0335541.s002.pdf]

S1b Table: Biomarker Subgroup Analysis

Measurement	Biomarker	Groups *				ANOVA (df = 3)	Post-Hoc P-value			
		**PD-NC**	**PD-MCI**	**HC-NC**	**HC-MCI**		**PD-NC vs PD-MCI**	**HC-NC vs HC-MCI**	**HC-NC vs PD-NC**	**HC-MCI vs PD-MCI**
**DaT Baseline SBR**	Left Caudate	1.90 (0.51)	1.85 (0.62)	3.00 (0.56)	2.91 (0.62)	**F = 71.46; p < .001**	0.975	0.742	**< .001**	**< .001**
	Right Caudate	1.84 (0.54)	1.89 (0.63)	2.94 (0.56)	2.89 (0.61)	**F = 64.5; p < .001**	0.976	0.970	**< .001**	**< .001**
	Left Putamen	0.76 (0.33)	0.77 (0.35)	2.19 (0.57)	2.05 (0.50)	**F = 171.6; p < .001**	0.998	0.293	**< .001**	**< .001**
	Right Putamen	0.78 (0.32)	0.80 (0.32)	2.20 (0.53)	2.04 (0.55)	**F = 161.8; p < .001**	0.995	0.141	**< .001**	**< .001**
	Left Anterior Putamen	1.25 (0.40)	1.21 (0.46)	2.61 (0.54)	2.53 (0.58)	**F = 142.3; p < .001**	0.987	0.782	**< .001**	**< .001**
	Right Anterior Putamen	1.24 (0.41)	1.28 (0.42)	2.64 (0.56)	2.54 (0.59)	**F = 133.3; p < .001**	0.976	0.654	**< .001**	**< .001**
**CSF Baseline** Conc. **(pg/ml)**	α-syn	1647.11 (921.41)	1478.68 (525.02)	1685.76 (730.31)	1758.37 (846.01)	F = 1.58; p = 0.194	-	-	-	-
	Aβ42	883.65 (339.67)	757.49 (311.73)	974.28 (326.53)	874.14 (399.06)	**F = 4.51; p = 0.004**	0.289	0.323	0.549	0.236
	t-tau	174.82 (70.16)	177.34 (58.96)	177.97 (64.56)	210.84 (94.39)	**F = 3.34; p = 0.018**	0.998	**0.036**	0.996	0.051
	p-tau	15.98 (6.07)	15.55 (5.81)	16.00 (5.88)	19.46 (11.05)	**F = 3.53; p = 0.016**	0.993	**0.039**	1.00	**0.024**
	NfL	95.31 (39.93)	133.58 (81.81)	91.10 (64.77)	109.50 (42.87)	**F = 3.44; p = 0.018**	0.064	0.514	0.996	0.283
**CSF Secondary** Conc. **(pg/ml)**	α-syn	1556.09 (706.16)	1492.01 (598.07)	1747.46 (650.26)	1844.39 (894.39)	**F = 3.18; p = 0.025**	0.972	0.850	0.518	**0.031**
	Aβ42	815.26 (385.28)	714.51 (328.86)	989.91 (297.88)	828.59 (371.42)	**F = 7.81; p < .001**	0.478	**0.029**	0.050	0.243
	t-tau	183.72 (73.33)	188.54 (82.56)	190.43 (75.81)	218.43 (106.97)	F = 2.01; p = 0.113	-	-	-	-
	p-tau	16.38 (6.31)	16.34 (7.23)	16.71 (6.56)	20.13 (11.47)	**F = 2.96; p = 0.033**	-	-	-	-
	NfL	117.11 (54.97)	193.86 (177.87)	99.79 (52.86)	118.42 (56.25)	**F = 7.03; p < .001**	**0.014**	0.807	0.888	**0.004**
**CSF** Rate of Change **(pg/(ml*mo))**	α-syn	-2.47 (17.84)	0.98 (13.89)	3.70 (25.42)	1.85 (17.84)	F = 0.89; p = 0.45	-	-	-	-
	Aβ42	1.40 (8.07)	0.92 (8.20)	-0.35 (9.33)	0.72 (10.20)	F = 0.40; p = 0.754	-	-	-	-
	t-tau	0.13 (1.30)	0.24 (1.23)	0.30 (1.63)	0.02 (1.22)	F = 0.55; p = 0.648	-	-	-	-
	p-tau	0.00 (0.09)	0.02 (0.10)	0.02 (0.14)	0.00 (0.10)	F = 0.32; p = 0.809	-	-	-	-
	NfL	0.57 (0.92)	1.78 (3.68)	0.18 (1.47)	0.20 (1.31)	**F = 4.98; p = 0.003**	0.116	1.00	0.872	**0.006**

Abbreviations: PD – Parkinson's Disease, HC – Healthy controls, NC – Normal Cognition; MCI – Mild Cognitive Impairment; DaT – DaT-SPECT; SBR – Striatal Binding Ratio; α-syn – Alpha-synuclein; Aβ42 – Beta-amyloid-42; t-tau – total-tau; p-tau – phosphorylated-tau-181; NfL – neurofilament light; Conc. - Concentration; pg – picogram; ml – millilitre; mo – month

All variables are reported with Mean (Standard Deviation). * Sample sizes are reported in Table 2.
