# Supplementary material for: Using machine learning for detection of Parkinson’s disease and mild cognitive impairment
Source: PLoS One. 2025 Nov 19;20(11):e0335541. doi: 10.1371/journal.pone.0335541 (PMC12629485; doi:10.1371/journal.pone.0335541)
Supplement: S2 Table — Abbreviations: SVM – support vector machine; RF – random forest; PD – Parkinson’s Disease; MCI – Mild Cognitive Impairment; α-syn – alpha-synuclein; Aβ42 – beta-amyloid-42; t-tau – total-tau; p-tau – phosphorylated-tau; NfL – neurofilament light; ACC – accuracy, AUC – area under the curve; KPA – kappa; SNS – sensitivity; SPC – specificity. (PDF) [file pone.0335541.s003.pdf]

S2 Table: Singular Models – Metric Performance for SVM and RF in HC-NC vs HC-MCI

	Metric	AUC	ACC	KPA	SNS	SPC
**α-syn**	**SVM**	0.62	68.61%	0.14	25.00%	90.58%
	**RF**	0.65	64.44%	0.15	34.17%	79.58%
**t-tau**	**SVM**	0.74	31.40%	-0.36	38.95%	25.42%
	**RF**	0.62	53.69%	-0.12	53.67%	33.34%
**NfL**	**SVM**	0.67	38.85%	-0.20	49.17%	30.00%
	**RF**	0.62	41.15%	-0.17	48.33%	35.00%
**DaT**	**SVM**	0.74	34.55%	-0.26	57.00%	15.83%
	**RF**	0.63	39.32%	-0.21	40.50%	38.33%
**p-tau**	**SVM**	0.76	31.95%	-0.32	46.11%	20.87%
	**RF**	0.68	35.37%	-0.28	42.78%	30.11%
**Aβ42**	**SVM**	0.80	29.51%	-0.38	37.78%	22.17%
	**RF**	0.73	33.66%	-0.30	42.78%	26.52%

Abbreviations: SVM – support vector machine; RF – random forest; PD – Parkinson's Disease; MCI – Mild Cognitive Impairment; α-syn – alpha-synuclein; Aβ42 – beta-amyloid-42; t-tau – total-tau; p-tau – phosphorylated-tau; NfL – neurofilament light; ACC – accuracy, AUC – area under the curve; KPA – kappa; SNS – sensitivity; SPC – specificity
