# Supplementary material for: Using machine learning for detection of Parkinson’s disease and mild cognitive impairment
Source: PLoS One. 2025 Nov 19;20(11):e0335541. doi: 10.1371/journal.pone.0335541 (PMC12629485; doi:10.1371/journal.pone.0335541)
Supplement: S3 Table — Abbreviations: SVM – support vector machine; RF – random forest; PD – Parkinson’s Disease; MCI – Mild Cognitive Impairment; α-syn – alpha-synuclein; Aβ42 – beta-amyloid-42; t-tau – total-tau; p-tau – phosphorylated-tau; NfL – neurofilament light; ACC – accuracy, AUC – area under the curve; KPA – kappa; SNS – sensitivity; SPC – specificity. (PDF) [file pone.0335541.s004.pdf]

**S3 Table: Singular Models – Metric Performance for SVM and RF in PD-NC vs PD-MCI**

| | Metric | AUC | ACC | KPA | SNS | SPC |
|---|---|---|---|---|---|---|
| **t-tau** | **SVM** | 0.66 | 39.00% | -0.07 | 12.22% | 79.17% |
| | **RF** | 0.61 | 40.67% | -0.08 | 22.22% | 68.33% |
| **DaT** | **SVM** | 0.65 | 38.00% | -0.11 | 15.00% | 72.50% |
| | **RF** | 0.62 | 41.00% | -0.11 | 27.78% | 65.83% |
| **Aβ42** | **SVM** | 0.65 | 37.59% | -0.07 | 12.22% | 79.09% |
| | **RF** | 0.65 | 38.97% | -0.12 | 25.56% | 60.91% |
| **α-syn** | **SVM** | 0.68 | 37.33% | -0.10 | 9.44% | 79.17% |
| | **RF** | 0.60 | 38.33% | -0.15 | 24.44% | 55.00% |
| **NfL** | **SVM** | 0.64 | 35.63% | -0.22 | 16.67% | 60.00% |
| | **RF** | 0.64 | 39.38% | -0.17 | 26.66% | 55.71% |
| **p-tau** | **SVM** | 0.75 | 31.79% | -0.16 | 11.67% | 68.00% |
| | **RF** | 0.68 | 34.29% | -0.21 | 26.11% | 49.00% |

*Abbreviations: SVM – support vector machine; RF – random forest; PD – Parkinson's Disease; MCI – Mild Cognitive Impairment; α-syn – alpha-synuclein; Aβ42 – beta-amyloid-42; t-tau – total-tau; p-tau – phosphorylated-tau; NfL – neurofilament light; ACC – accuracy, AUC – area under the curve; KPA – kappa; SNS – sensitivity; SPC – specificity*
