# Supplementary material for: Using machine learning for detection of Parkinson’s disease and mild cognitive impairment
Source: PLoS One. 2025 Nov 19;20(11):e0335541. doi: 10.1371/journal.pone.0335541 (PMC12629485; doi:10.1371/journal.pone.0335541)
Supplement: S4 Table — Abbreviations: SVM – support vector machine; RF – random forest; HC – Healthy Controls; MCI – Mild Cognitive Impairment; Aβ42 – beta-amyloid-42; p-tau – phosphorylated-tau; ACC – accuracy, AUC – area under the curve; KPA – kappa; SNS – sensitivity; SPC – specificity. (PDF) [file pone.0335541.s005.pdf]

S4 Table: Combined Models – Metric Performance for SVM and RF in HC-NC vs HC-MCI

	Metric	AUC	ACC	KPA	SNS	SPC
**DaT + p-tau**	**SVM**	0.56	50.00%	0	0%	100%
	**RF**	0.48	70.00%	0.4	40%	100%
**DaT + p-tau + Aβ42**	**SVM**	0.48	50.00%	-0.05	28.57%	66.67%
	**RF**	0.71	62.50%	0.21	42.86%	77.78%
**DaT + Aβ42**	**SVM**	1	0.00%	-0.92	0.00%	0.00%
	**RF**	1	30.00%	-0.52	50.00%	0.00%

Abbreviations: SVM – support vector machine; RF – random forest; HC – Healthy Controls; MCI – Mild Cognitive Impairment; Aβ42 – beta-amyloid-42; p-tau – phosphorylated-tau; ACC – accuracy, AUC – area under the curve; KPA – kappa; SNS – sensitivity; SPC – specificity
