# Supplementary material for: Using machine learning for detection of Parkinson’s disease and mild cognitive impairment
Source: PLoS One. 2025 Nov 19;20(11):e0335541. doi: 10.1371/journal.pone.0335541 (PMC12629485; doi:10.1371/journal.pone.0335541)
Supplement: S5 Table — Abbreviations: SVM – support vector machine; RF – random forest; MCI – Mild Cognitive Impairment; PD – Parkinson’s; α-syn – alpha-synuclein; t-tau – total-tau; p-tau – phosphorylated-tau; NfL – neurofilament light; ACC – accuracy, AUC – area under the curve; KPA – kappa; SNS – sensitivity; SPC – specificity. (PDF) [file pone.0335541.s006.pdf]

S5 Table: Combined Models – Metric Performance for SVM and RF in PD-NC vs PD-MCI

	Metric	AUC	ACC	KPA	SNS	SPC
**DaT + α-syn + t-tau**	**SVM**	0.63	63.64%	0	100.00%	0.00%
	**RF**	0.66	54.55%	0.23	28.57%	100.00%
**DaT + NfL + t-tau**	**SVM**	0.5	57.14%	0	100.00%	0.00%
	**RF**	0.5	57.14%	0.22	25.00%	100.00%
**DaT + NfL**	**SVM**	0.5	57.14%	0	100.00%	0.00%
	**RF**	0.75	57.14%	0	100.00%	0.00%
**DaT + t-tau**	**SVM**	0.5	37.50%	0	100%	0%
	**RF**	0.57	62.50%	0.25	66.67%	60.00%
**DaT + p-tau**	**SVM**	0.5	60%	0	100%	0%
	**RF**	0.75	25.00%	-0.43	0.00%	50.00%
**DaT + α-syn**	**SVM**	0.5	40%	0	100%	0%
	**RF**	0.65	40%	0	100%	0%

Abbreviations: SVM – support vector machine; RF – random forest; MCI – Mild Cognitive Impairment; PD – Parkinson's; α-syn – alpha-synuclein; t-tau – total-tau; p-tau – phosphorylated-tau; NfL – neurofilament light; ACC – accuracy, AUC – area under the curve; KPA – kappa; SNS – sensitivity; SPC – specificity
