# Supplementary material for: Using machine learning for detection of Parkinson’s disease and mild cognitive impairment
Source: PLoS One. 2025 Nov 19;20(11):e0335541. doi: 10.1371/journal.pone.0335541 (PMC12629485; doi:10.1371/journal.pone.0335541)
Supplement: Attachment — Submitted filename: Reviewer_Responses_09302025.pdf [file pone.0335541.s008.pdf]

| Comments by Academic Editor | |
|---|---|
| # | |
| 1 | Please ensure that your manuscript meets PLOS ONE's style requirements, including those for file naming. The PLOS ONE style templates can be found at https://journals.plos.org/plosone/s/file?id=wjVg/PLOSOne_formatting_sample_main_body.pdf and https://journals.plos.org/plosone/s/file?id=ba62/PLOSOne_formatting_sample_title_authors_affiliations.pdf |
| | The manuscript was reformatted and the files were renamed to comply with the style requirements. |
| | |
| 2 | Please note that PLOS One has specific guidelines on code sharing for submissions in which author-generated code underpins the findings in the manuscript. In these cases, we expect all author-generated code to be made available without restrictions upon publication of the work. Please review our guidelines at https://journals.plos.org/plosone/s/materials-and-software-sharing#loc-sharing-code and ensure that your code is shared in a way that follows best practice and facilitates reproducibility and reuse. |
| | Code is available at https://zenodo.org/records/17195714?token=eyJhbGciOiJIUzUxMiJ9.eyJpZCI6IjQ2NzlhYzU1LWVmNjItNDc2Zi05OTQ4LWUwNjhkMjAxNjJlYyIsImRhdGEiOnt9LCJyYW5kb20iOiIwODg2ZjQ2MDQzYzFlZDMzNDA2NjgwNDQyNzFjNGU2MyJ9.11i_IRH_6RlcMpfT7JftVXTcRont4_JILk4_Er7gcHTRAfWxLDOoJJzTYj25NxeMEfRazKubunClCH7mUOiJVg. The availability of the code will change from restricted to public once the manuscript has been published. |
| | |
| 3 | Thank you for stating in your Funding Statement:<br>"A-G.P.D. is supported by the Queen Elizabeth II Graduate Scholarship in Science & Technology (https://www.sgs.utoronto.ca/awards/queen-elizabeth-ii-graduate-scholarship-in-science-technology/), the Krembil Brain Institute (https://www.uhn.ca/Krembil), and the University of Toronto School of Graduate Studies (https://www.sgs.utoronto.ca/). A.P.S. is supported by the Canadian Institute of Health Research (CIHR) (PJT-173540) (https://cihr-irsc.gc.ca/e/193.html) and the Krembil-Rossy Chair program (https://rossy-foundation.org/; https://www.uhn.ca/Krembil). These funders did not play any role in the study design, data collection and analysis, decision to publish, or preparation of the manuscript."<br><br>Please provide an amended statement that declares *all* the funding or sources of support (whether external or internal to your organization) received during this study, as detailed online in our guide for authors at http://journals.plos.org/plosone/s/submit-now. Please also include the statement "There was no additional external funding received for this study." in your updated Funding Statement.<br>Please include your amended Funding Statement within your cover letter. We will change the online submission form on your behalf. |
| | The amended funding statement is in the cover letter and here:<br>A-G.P.D. is supported by the Queen Elizabeth II Graduate Scholarship in Science & |

| | |
|---|---|
| | Technology (https://www.sgs.utoronto.ca/awards/queen-elizabeth-ii-graduate-scholarship-in-science-technology/), the Krembil Brain Institute (https://www.uhn.ca/Krembil), and the University of Toronto School of Graduate Studies (https://www.sgs.utoronto.ca/). A.P.S. is supported by the Canadian Institute of Health Research (CIHR) (PJT-173540) (https://cihr-irsc.gc.ca/e/193.html) and the Krembil-Rossy Chair program (https://rossy-foundation.org/; https://www.uhn.ca/Krembil). These funders did not play any role in the study design, data collection and analysis, decision to publish, or preparation of the manuscript. There was no additional external funding received for this study. |
| | |
| 4 | Thank you for uploading your study's underlying data set. Unfortunately, the repository you have noted in your Data Availability statement does not qualify as an acceptable data repository according to PLOS's standards.<br>At this time, please upload the minimal data set necessary to replicate your study's findings to a stable, public repository (such as figshare or Dryad) and provide us with the relevant URLs, DOIs, or accession numbers that may be used to access these data. For a list of recommended repositories and additional information on PLOS standards for data deposition, please see https://journals.plos.org/plosone/s/recommended-repositories. |
| | All data used in this study, as well as a data dictionary, are free and publicly available at the PPMI website (https://www.ppmi-info.org/access-data-specimens/download-data), upon an online application, the signature of the Data User Agreement and of the publications policies. For any questions on the PPMI Intellectual Property (IP) Policy or applying for an exception to the PPMI IP Policy to ppmi@michaeljfox.org. We declare that we did not have any special access privileges that others would not have when attempting to access the data from PPMI. |
| | |
| 5 | Please include captions for your Supporting Information files at the end of your manuscript, and update any in-text citations to match accordingly. Please see our Supporting Information guidelines for more information: http://journals.plos.org/plosone/s/supporting-information. |
| | The manuscript file has been updated to include captions for supporting information files. |
| | |
| 6 | If the reviewer comments include a recommendation to cite specific previously published works, please review and evaluate these publications to determine whether they are relevant and should be cited. There is no requirement to cite these works unless the editor has indicated otherwise. |
| | N/A |
| | |
| Comments by Reviewer #1 | |
| # | |
| 1 | In Table 2, the descriptions of total-tau and phosphorylated-tau-181 appear to be swapped. Please verify and correct this, and confirm that the analyses themselves were not affected. |
| | This has been corrected. |
| | |
| 2 | Although authors reported 10-fold cross-validation with a 70/30 train test split, the instances of perfect accuracy suggest possible overfitting. It would strengthen the findings to consider more rigorous strategies such as nested or repeated cross-validation, and to report confidence |

| | |
|---|---|
| | intervals for the performance metrics. |
| | A statement on the possibility of overfitting and the use of more rigorous techniques in the future had been added. Confidence intervals were available for the 100% accuracy results, so these were added into the table reported these results. |
| | |
| 3 | At present, only SVM and Random Forest have been applied. Including, or at least benchmarking against, an additional method such as gradient boosting or penalized regression would make the results more robust and provide better context for the reader. |
| | We benchmarked against the accuracy of clinical diagnosis, which is approximately 84%, and used a threshold of 80% as a uniform metric (page 13). Since both SVM and RF models for detecting PD achieved over these thresholds, this shows that regardless of the specific technique used, a machine learning approach can be an improvement on the current clinical methods. Additional context was provided in the Discussion section. |
| | |
| 4 | The models also did not reliably classify MCI, so it would be best to frame the conclusions more cautiously and provide a discussion of possible biological or methodological reasons for this outcome. |
| | More information on the models for MCI and possibilities for poor performance was added in the Discussion section (pages 22 – 24). |
| | |
| 5 | The manuscript states that an alpha value of 0.05 was used for all tests. Please clarify if this refers to the p-value threshold, and provide more detail on how statistical tests were applied, particularly with regard to assumptions of normality and variance. |
| | Additional information was provided on pages 6 – 7. |
| | |
